# Neurasites^®^—A Standardized Plant Extract in the Treatment and Prevention of Migraine Attacks

**DOI:** 10.3390/jcm13123364

**Published:** 2024-06-07

**Authors:** Emilia Furdu Lunguț, Claudia Antal, Suzana Turcu, Valentin-Nicolae Varlas, Alexandru George Filipescu, Irina Balescu, Nicolae Bacalbașa, Gabriel-Petre Gorecki

**Affiliations:** 1 Department of Neurology, Faculty of Medicine, “Titu Maiorescu” University, 031593 Bucharest, Romania; emilia.furdu@spcf2.ro; 2 Department of Neurology, CF2 Clinical Hospital, 011464 Bucharest, Romania; 3 2gether NHS Foundation Trust for Gloucestershire, Gloucestershire GL3 4AW, UK; claudiaantal@yahoo.com; 4 Romanian Academy, “Francisc I. Rainer” Institute of Anthropology, 050711 Bucharest, Romania; suzana.turcu@antropologia.ro; 5 Department of Obstetrics and Gynecology, “Carol Davila” University of Medicine, 050474 Bucharest, Romania; valentinvarlas@yahoo.com (V.-N.V.); alexandrufilipescu@yahoo.com (A.G.F.); 6 Department of Obstetrics and Gynecology, Filantropia Clinical Hospital, 011132 Bucharest, Romania; 7 Department of Obstetrics and Gynecology, Elias Emergency Clinical Hospital, 011461 Bucharest, Romania; 8 Department of General Surgery, “Carol Davila” University of Medicine, 050474 Bucharest, Romania; irina_balescu206@yahoo.com; 9 Department of Visceral Surgery, ‘Carol Davila’ University of Medicine and Pharmacy, 050474 Bucharest, Romania; 10 Department of Visceral Surgery, Center of Excellence in Translational Medicine “Fundeni” Clinical Institute, 022328 Bucharest, Romania; 11 Department of Visceral Surgery, Center of Digestive Diseases and Liver Transplantation, Fundeni Clinical Institute, 022328 Bucharest, Romania; 12 Department of Anesthesia and Intensive Care, “Titu Maiorescu” University, Faculty of Medicine, 031593 Bucharest, Romania; Gabriel.gorecki@prof.utm.ro; 13 Department of Anesthesia and Intensive Care, CF2 Clinical Hospital, 011464 Bucharest, Romania

**Keywords:** Neurasites^®^, standardized extract, plant, migraine, treatment, prevention

## Abstract

**Background/Objectives:** Migraine is one of the most common diseases in highly developed countries, being even more common than diabetes and asthma. Migraines can affect emotional, social, and physical wellbeing as well as professional life. The most common symptoms are severe headaches associated with nausea, vomiting, photophobia and sonophobia, difficulty concentrating, sensitivity and emotional disorders. Many studies have been published to establish the best migraine-management drugs, but not many of them refer to plant extracts, which have been given more attention by patients lately. Among these generically called herbal medicines, the effect of tussilago hybrida standardized extract has been studied since the early twenties. This stands as the fundamental component of Neurasites^®^ and the reason for research on materials and methods, results on treatment schemes for diminishing migraine attack features, as well as migraine prevention. **Methods:** There are two directions of research (herbal and placebo medicine) considered to be of interest due to the actual trend toward natural medicine and against chemicals and associated drugs. For quantitative research, the research tool used was that of the Neurasites^®^ Questionnaire Survey (NQS). **Results:** The obtained results prove the efficacy of treatment by reducing the duration of headache attacks, diminishing pain intensity and decreasing the frequency of migraine episodes. **Conclusions:** Further research development should focus on other dosages and treatment schemes and on other similar natural products to be used in migraine attack treatment.

## 1. Introduction

Migraine is one of the most common diseases in highly developed countries, being even more common than diabetes and asthma. It is a neurological disorder that ranks second worldwide [1,2,3], and it is responsible for more disabilities than all other neurological disorders combined [4,5]. Worldwide, about 15% of women and men suffer from migraine. However, many people have a false image of migraine, and there are many prejudices, such as, for example, the fact that only women have migraines or that people who suffer from migraines are very sensitive. Migraines can affect emotional, social, and physical wellbeing as well as professional life.

The solution to the migraine problem is studied in various scientific research studies in terms of causes identified by medical staff or expressed by patients. Thus, some relevant aspects are envisaged, as follows in the next paragraphs.

Mangrum R et al. [6] pointed out a list of rank-ordered benefits for preventive migraine, such as the level of daily physical and intellectual demands, while A-Hassany L et al. [7] evidence the influence of successful prophylaxis duration and the effects of therapy discontinuation.

There is a relevant paper published by Tzankova V. et al. [8] that points out aspects of pharmacological prevention of migraine and mentions vitamins and herbal supplements for migraine prevention, out of which butterbur, a commercial product with a minimum of 15% petasins, can be highlighted.

Silva Neto et al. [9] point out the three pillars of migraine treatment: disease and prevention treatment, acute treatment and patient education. Attention is given to the latest one, mainly to the weekly duration of physical exercises carried out in an organized setting.

A systematic review of pharmacological treatments is presented by Lampl C. et al. [10], the focus being on migraine prophylaxis in adults. The conclusion is that most individuals pointed toward CGRP(r)mAbs as a first-line option. The same trend is evidenced by Bentivegna E. et al. [11], who consider calcitonin gene-related peptide (CGRP) receptor antagonists as a turning point in migraine prophylaxis.

Puledda F et al. [12,13] present aspects of up-to-date therapeutic agents for the treatment of migraine, including molecules targeting CGRP, serotonin 5-HT_1F_ receptor agonists and non-invasive neuromodulation, as options for both acute and preventive treatment.

A study on Rimegepant [NurtecR ODT (USA); Vydura R (EU)] used in acute and preventive treatment of migraine was presented by Blair H.A. [14]. This is also a CGRP antagonist, and it was found to be more effective than placebo in migraine treatment. The conclusion was that it was well tolerated by patients, and the majority of adverse effects were not severe.

The most common symptoms of migraine are severe headaches associated with nausea, vomiting, photophobia and phonophobia, difficulty concentrating, sensitivity and emotional disorders [15]. Although the level of suffering is extremely high and the quality of life is severely affected, most patients endure pain in silence; moreover, almost 70% of migraine patients are not treated sufficiently.

There are six types of migraine according to the third edition of the International Classification of Headache Disorders (ICHD-3) [16,17], as follows: migraine without aura, migraine with aura, chronic migraine, complications of migraine, probable migraine and episodic syndromes that may be associated with migraine.

In primary care, an ID Migraine Screener [6,18] can be used as a tool for assessing migraine. It screens for migraine-associated features, which are photophobia, functional impairment and nausea. The result is positive for migraine if, at least, two of these features are “pinned”.

**Figure 1:**
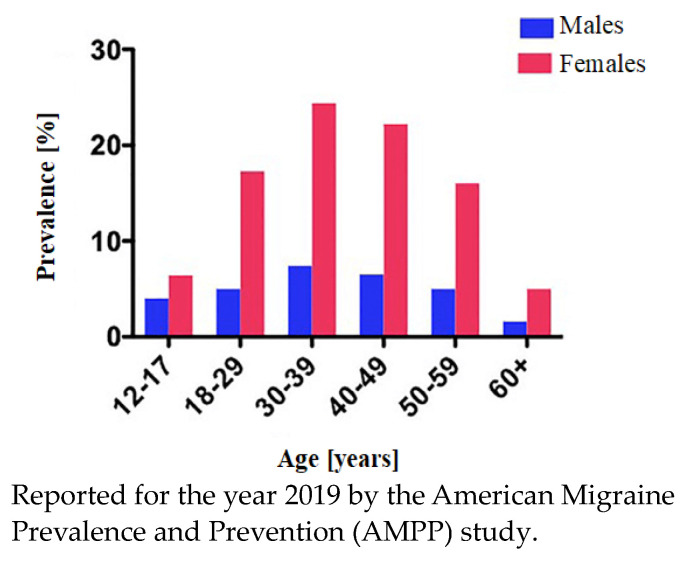
Prevalence of migraine by age and sex [20].

There are many studies on the prevalence of migraine over an extended period of time, for example, the period between 2005 [19] and 2019 [20]. It is observed that people in their 20s up to their 50s experience migraine attacks (Figure 1). When it comes to sex, females are predominant and much more exposed to migraine than males.

In [1,21], the acute care treatments for the step–care approach are mentioned. They are named first–line, second–line and third–line.

First-line medication includes non-steroidal anti-inflammatory drugs (NSAIDs), with strong support from acetylsalicylic acid, ibuprofen and diclofenac potassium [22,23].

Second-line medication mainly refers to triptans, taken early during an attack when the headache is still mild. No evidence supports the use of triptans during the aura phase of a migraine attack [24,25].

Third-line medication is used if triptan medication fails or it is not indicated for use. Then, medication with ditans or gepants could be used, but their availability is very limited. [26,27].

Adjunct medication consists of antiemetics (domperidone and metoclopramide) and is targeted against the nausea and/or vomiting accompanying migraine attacks.

The American Federation of Neurological Societies highlights second-choice drugs in migraine prophylaxis (evidence of efficiency but less effectiveness or more side effects than drugs of first choice) [28]. Petasites, which is a standardized extract from Petasites hybridus (the butterbur plant),is to be noticed. It combines with magnesium and Q10 coenzyme to form Neurasites^®^ (a standardized plant extract).

The study presented by [28] evidences that Neurasites^®^ administration for two months determined a 60% reduction in migraine frequency and a 50% reduction in migraine intensity.

This paper briefly shows the importance of migraine treatment and prevention given the spread of migraine disease throughout the population worldwide. There are different treatment drugs and schemes presented in the References, but not many of them refer to plant extracts, which have been given more attention by patients lately.

The next chapters of this article present the materials and methods used, the results of our research on Neurasites^®^ treatment schemes for diminishing migraine attack features and for migraine prevention, a discussion and our conclusions based on the obtained results.

## 2. Materials and Methods

It should be mentioned that first-line prophylactic treatment includes beta-blocker medicines, more specifically metoprolol (daily dose between 50 and 200 mg) and propranolol (daily dose between 40 and 240 mg). Other beta-blockers, like flunarizine and calcium channel blockers, are not usually used by doctors in the hospitals where this study was conducted.

Then, there is the antiepileptic treatment, which consists in the administration of the following drugs: valproic acid, in a daily dose of at least 600 mg, and topiramate, in a daily dose of 25–100 mg.

The scope of this study is to show the impact of Neurasites^®^ in the treatment and prevention of migraine attacks as a vitamin and herbal supplement.

Neurasites^®^ (derived from a genus of flowering plants in the sunflower family, Asteraceae, commonly referred to as butterbur)—150 mg with a minimum of 7.5% sesquiterpene (S-petasin, which supports the normal functioning of blood vessels)—acts to inhibit vasodilation and cyclooxygenase so as to reduce the duration of migraine attacks. The frequency of the attacks is reduced through the regulating effect on calcium channels and by inhibiting the synthesis of leukotri, thus resulting in a decrease in the intensity of pain [29].

In association with this standardized plant extract, the following supplements are to be prescribed.

Magnesium oxide monohydrate (460 mg) and magnesium (276 mg). Limited evidence does indicate their efficiency in migraine prophylaxis. Magnesium’s role can be hypothesized in migraine pathogenesis through the modulation of 5-HT neurotransmission, the nitric oxide pathway, and mitochondrial oxidative phosphorylation. It may also initiate and propagate CSD. Moreover, it is thought to interrupt the NMDA receiver function and regular glutamate uptake into astrocytes. It also downregulates the activity of matrix metalloproteinase-2, which may be involved in blood–brain barrier disruption in migraine [30,31].

Antioxidant coenzyme Q10 (50 mg) for reducing the level of pain-related protein (CGRP) and vitamin D (2000 UI) for increasing serotonin levels are also recommended [32,33].

**Figure 2:**
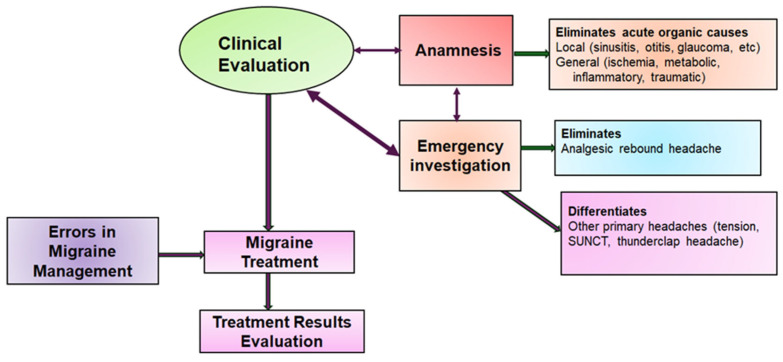
Algorithm for migraine diagnosis.

The algorithm scheme for migraine diagnosis is shown in Figure 2. The main steps included are as follows: clinical evaluation; treatment; and, not least, evaluation after treatment.

The sample initially included in this study considered adults aged 18 to 65 years with a diagnosis of migraine per the International Classification of Headache Disorders criteria. Key inclusion criteria were a history of migraine for at least 12 months, with no more than 14 headache days per month, including 2 or more migraine days in the 3 months before screening.

Applying these “filters” resulted in a sample of patients for our study, comprising a total of 85 eligible individuals, of whom 64 were women and 21 were men. Two groups were formed, each consisting of both women and men between the ages of 20 and 50 years, suffering from migraine, migraine with aura and chronic/complicated migraine.

For quantitative research, the investigation method was used, and the research tool was a questionnaire.

**Figure 3:**
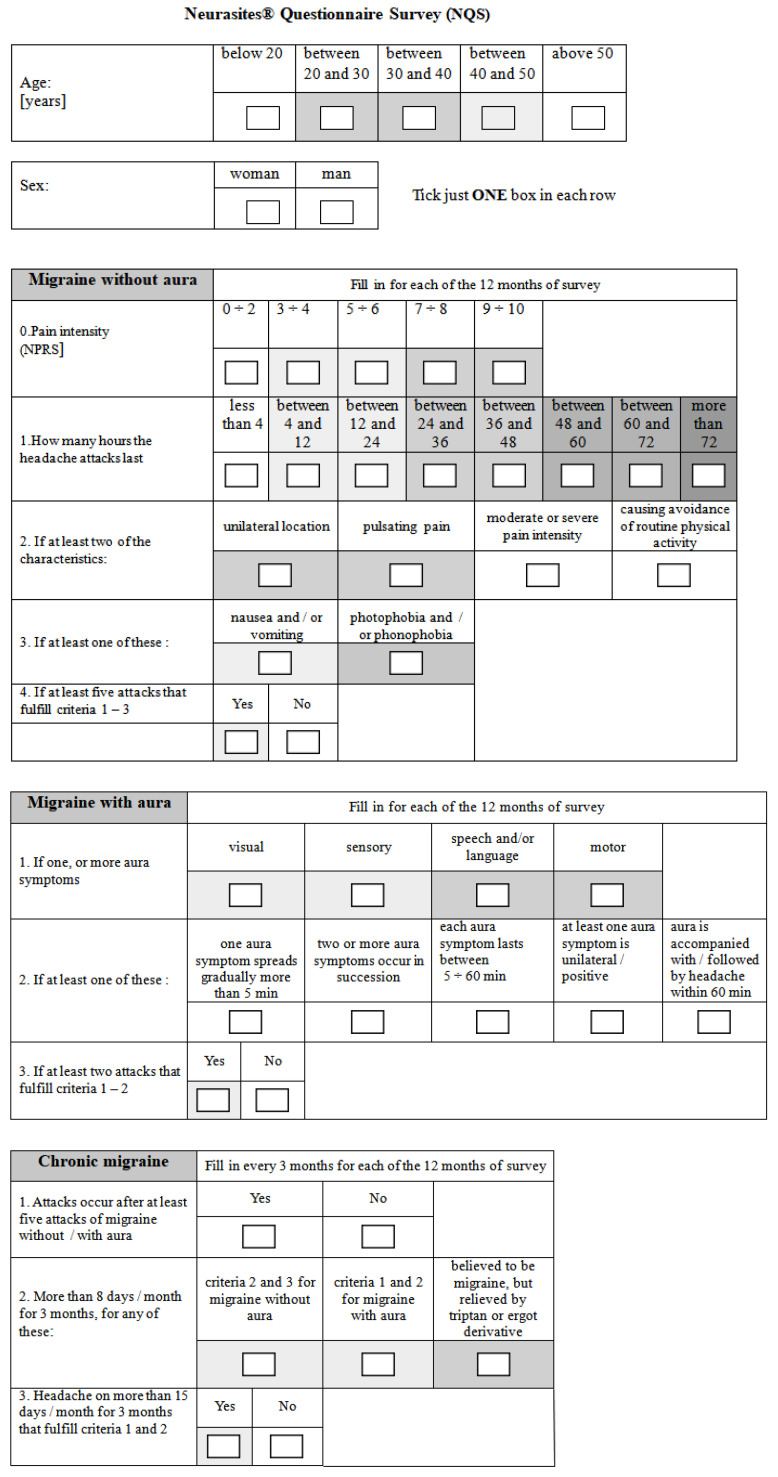
Neurasites^®^ Questionnaire Survey (NQS).

Patients were asked to complete a questionnaire (either classically, handwritten, or electronic, eDiary). The concept of this questionnaire is new, but it still considers both the HURT questionnaire (Headache Under-Response to Treatment) [34] and the ICHD-3 diagnostic criteria for primary headache disorders [1,17]. It is called the Neurasites^®^ Questionnaire Survey (NQS) and is presented in Figure 3.

Pain intensity was evaluated by the Numeric Pain Rating Scale (NPRS) [35].

Two directions and focuses of research (herbal and placebo medicine) were considered to be of interest due to the ancient, but still very actual, trend toward natural medicine and against chemicals and associated drugs (pharmacological drugs).

Patients were divided into two groups. The first group, made up of 15 patients (both women and men), received placebo medication for 12 months. The second one, made up of 70 patients (both women and men), received Neurasites^®^-based treatment.

For the second group, permanent treatment with Neurasites^®^—1 tablet/day—was established (in the morning, during a meal, as prophylactic treatment for 12 months). Patients diagnosed with migraine with aura were also given 1 tablet of Imigran 50 mg during the aura phase. Patients with chronic/complicated migraine associated Neurasites^®^ with Topiramat 25 mg or 50 mg, 2 tablets/day, in the morning and the evening, or only in the evening, depending on the severity and intensity of the seizures (see classification of migraine types in [1,17]).

**Figure 4:**
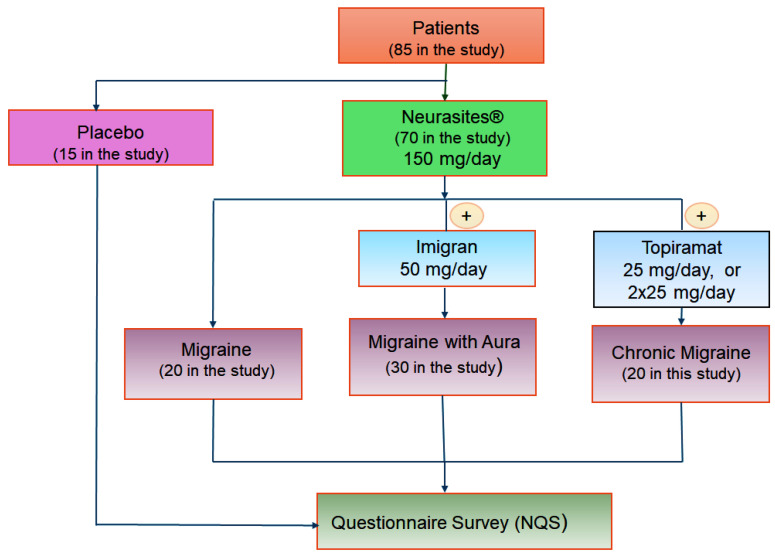
Treatment scheme based on Neurasites^®^.

After 12 months, treatment was stopped. The representation of the treatment scheme followed by the patients in the sample study is shown in Figure 4.

## 3. Results

The total duration of this study was 12 months, with 12 scheduled visits—at the end of each month of the survey. The data from the questionnaire were summarized each month after the visit. Once all patients completed the whole survey period, these data were processed and analyzed.

As an example, in patients who had treatment with Neurasites^®^, the results on headache duration, attack frequency and pain intensity are shown in Table 1. The data represent the arithmetic mean of the numbers ticked for each month (from the first month, M1, up to the last month, M12) by each patient.

**Figure 5:**
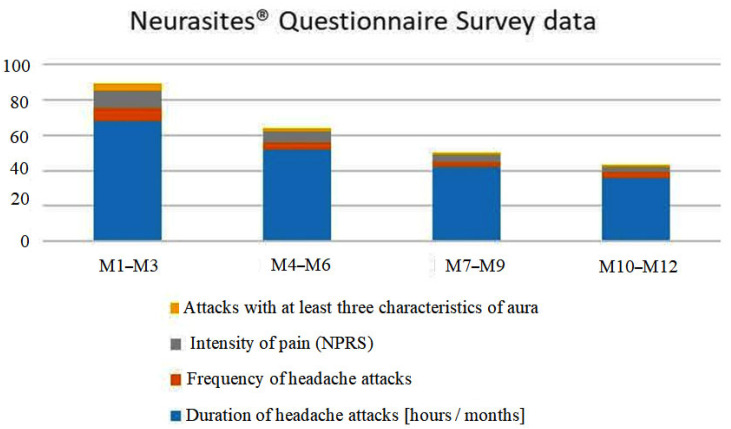
Questionnaire survey data plot.

A plot of the questionnaire survey data is shown in Figure 5.

Of the patients in the second group, treated with placebo, only 3% experienced a slight improvement regarding the duration and intensity of migraine attacks.

## 4. Discussion

The results indicate that all patients in our study reported a significant decrease in the number of hours of pain caused by migraine by more than 45% compared to the duration prior to the beginning of the treatment. Following that, after 12 months, 48% of them would notice a significant decrease in the intensity of the pain.

**Figure 6:**
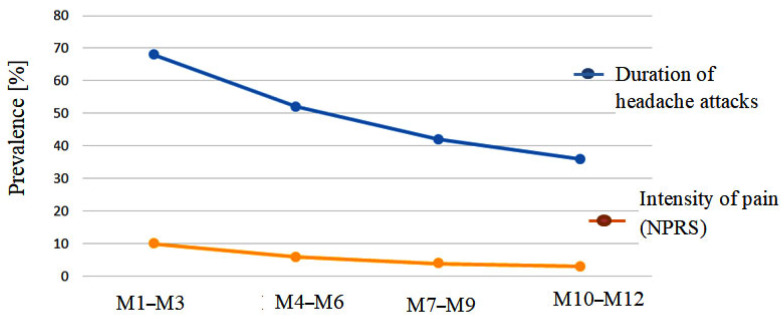
Graphical representation of NQS data.

Also, it can be noticed (see Figure 6) that there is a relevant decrease in headache duration in the first 6 months of the treatment (by about 23%), while in the second half of the survey, there is a lower rate of decrease (about 14%). Similar results, when referring to the efficacy of Neurasites^®^ treatment during the 12-month survey, are to be noticed for the intensity of pain.

Attacks with at least three characteristics of aura had a relevant decrease during the first six months of treatment. They proved to be constant in number for the second part (M7–M12) of the survey period.

After 12 months, 84% of subjects reported a decrease in the frequency of migraine crises by more than 55%. Relevant migraine attacks mostly reappeared in special situations such as sleep deprivation, excessive alcohol consumption, mental stress and conflict situations.

It is to be noticed that all questions in the Neurasites^®^ Questionnaire Survey are strictly intended for scientific purposes. The questionnaire is anonymous, and the answers are confidential and not related to the patients. Moreover, patients can give up and not take part in the survey whenever they consider it appropriate.

## 5. Conclusions

The prevention of migraine attacks becomes an important standard to achieve, given the 237 social, professional, familial and economic implications of this pathology.

The first-line prophylactic treatment is with beta-blockers: metoprolol (daily dose between 50 and 200 mg) and propranolol (daily dose between 40 and 240 mg). The antiepileptic treatment consists in administering valproic acid (in a daily dose of at least 600 mg) and topiramate (in a daily dose of 25–100 mg).

There are different treatment drugs and schemes for migraine treatment and prevention, but few refer to plant extracts.

The results presented in this paper regarding Neurasites^®^ treatment schemes for diminishing migraine attack features and for migraine prevention are promising. They prove the reduction in duration of headache attacks, the diminution of pain intensity and, not least, the decrease in the frequency of migraine episodes. The efficacy of this extract is obviously higher than that of the placebo treatment.

It is important to notice that Neurasites^®^ is a standardized plant extract (butterbur), so, being natural, it is estimated (as most natural drugs available are) not to have toxic secondary effects on the patient’s body. This aspect is important as, mainly, migraine affects relatively young people, who must not be exposed to toxicity.

Due to the fact that it is a natural extract, Neurasites^®^-based treatment is accepted and trusted by patients. Not least, this product is available at a reasonable price for the majority of patients, which is why this treatment is so very affordable.

Further research development should focus on other dosages and treatment schemes with Neurasites^®^ (50 mg/day; 100 mg/day) and on other similar natural products to be used in the treatment and prevention of migraine attacks.

## Figures and Tables

**Table 1:** Neurasites^®^ Questionnaire Survey data.

Questionnaire Data	Months [M]			
	M1–M3	M4–M6	M7–M9	M10–M12
Duration of headache attacks [hours/months]	68	52	42	36
Frequency of headache attacks	7	4	3	3
Intensity of pain (NPRS)	10	6	4	3
Attacks with at least three characteristics of aura	4	2	1	1

## Data Availability

Supplemental data are available upon reasonable request.
